# Promises and challenges of AI-enabled methods for myocardial characterisation in cardiovascular magnetic resonance

**DOI:** 10.3389/fcvm.2026.1638861

**Published:** 2026-01-30

**Authors:** N. McWilliams, M. Varela, G. Joy

**Affiliations:** 1 Cardiovascular Research Institute, Cardiovascular Clinical Academic Group, City St George's University of London, London, United Kingdom; 2 Cardiology Department, St George's University Hospitals NHS Foundation Trust, London, United Kingdom; 3 National Heart & Lung Institute, Imperial College London, United Kingdom

**Keywords:** artificial intelligence, cardiac magnetic resonance, diffusion tensor imaging, radiomics, tissue characterisation

## Abstract

Cardiac magnetic resonance (CMR) tissue characterisation is central to the diagnosis and risk stratification of myocardial disease. However, for certain techniques tissue characterisation CMR is limited by reliance on contrast agents, sensitivity to motion, prolonged acquisition times, and time- and labour-intensive image reconstruction and analysis. Artificial intelligence (AI) has emerged as a promising approach to address these challenges by enhancing and accelerating multiple stages of the CMR workflow. Deep learning methods can automate LGE segmentation, improve motion correction and image reconstruction for parametric mapping, and enable contrast-free characterisation of scar by exploiting native CMR signals, including myocardial motion and native T1 mapping. AI has also accelerated emerging techniques such as cardiac magnetic resonance fingerprinting and diffusion tensor imaging. In addition, radiomics and deep learning–based feature extraction offer the potential to derive high-dimensional tissue phenotypes and risk markers beyond those identifiable by expert clinicians. Despite these advances, translation remains limited by access to large-scale, heterogeneous training data, alongside concerns over generalisability, fairness, and interpretability, as well as barriers to regulatory approval and clinical deployment. In this mini-review, we summarise recent developments in AI-enabled myocardial tissue characterisation using CMR, highlighting both the promises and challenges for clinical translation.

## Introduction

### Clinical importance of tissue characterisation CMR

The role of Cardiac MRI (CMR) in guiding care has expanded in recent years due to its unique ability to characterise key myocardial disease processes. Central to the diagnostic power of CMR is the characterisation of focal fibrosis (scar) by late gadolinium enhancement (LGE) which employs gadolinium-based contrast agent (GBCA) (1, 2). LGE transmurality due to myocardial infarction was thought to predict recovery of function through revascularisation (3) although this paradigm has recently been challenged (4). In hypertrophic cardiomyopathy (HCM), a high burden of LGE (defined as >15% of myocardium) influences risk stratification (5). In non-ischaemic cardiomyopathy (NICM), specific scar patterns may be suggestive of underlying genetic substrate (6). Furthermore, the presence of LGE appears to predict arrhythmic risk (7), and may therefore guide the implantation of devices in the future (8). Other myocardial processes can be characterised using quantitative parametric mapping [T1, T2/T2* and extracellular volume (ECV)], which plays a key role in phenotyping myocardial disease and guiding treatment (9). T1 maps can quantify myocardial diffuse fibrosis in multiple diseases and infiltration such as amyloid deposition and detect storage disorders such as Fabry's disease (9). Post-contrast T1 mapping allows calculation of the extracellular volume fraction (ECV) (10) which is particularly useful in detecting cardiac amyloidosis and monitoring its response to treatment (10). T2 maps allows detection of active inflammation in myocarditis, cardiac sarcoidosis, and Takotsubo cardiomyopathy (9, 11, 12). T2* mapping is the standard method for detecting and quantifying myocardial iron overload, and guiding chelation therapy (13).

**Table 1:** Summary of different AI techniques, how they are applied to tissue characterisation advantages and potential challenges.

Domain	AI techniques	CMR applications	Key advantages and challenges
Late gadolinium enhancement segmentation	Convolutional Neural Networks (CNNs)	- Automated scar segmentation (ischaemic & non-ischaemic) scar burden quantification in hypertrophic cardiomyopathy (HCM) and ischaemic cardiomyopathy (ICM)	- AI LGE segmentation - Reduce inter-/ intra-observer variability - Enables rapid analysis at scale - Limited by a lack of standardized ground truth & large scale external validation
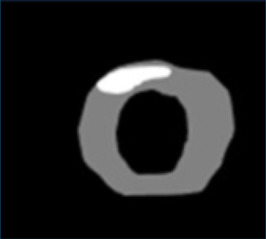	Fully Convolutional Networks (FCNs)		
	Long Short-Term Memory Recurrent Neural Networks (LSTM-RNNs)		
	Autoencoders		
	Gradient weighted Class activation mapping (GradCAM) interpretability/weak-supervision method		
	Vision Foundation Models self-supervised large-scale pretraining		
Synthetic Post-contrast Imaging	Generative Adversarial Networks (GANs)	- Contrast-free detection of focal fibrosis in HCM, myocardial infarction (MI)	Virtual LGE
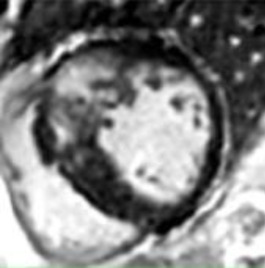	- Virtual Native Enhancement (VNE) - Cine-Generated Enhancement (CGE)		- Improves accessibility (renal failure, allergy) only in proof-of-concept stage - Needs validation in multiple diseases, scanners, sequences and externally on diverse clinical datasets
	Recurrent neural networks long-short term memory		
Parametric Mapping (T1/T2/T2*/ECV)	CNN-based motion correction (MOCO)	-Motion correction in mapping acquisitions	AI MOCO and reconstruction
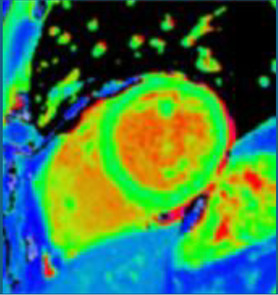	End-to-end DL reconstruction frameworks	- Rapid 3D whole-heart T1/T2 mapping - Artefact suppression - Virtual ECV mapping from native T1 input	- Improves reproducibility & quantitative accuracy and reduces reconstruction times - Needs to be applied in-line across scanners and vendors for widescale adoption
	Generative Adversarial Networks		vECV
			- Proof-of concept stage but current risks include missing of focal mapping lesions and GAN based hallucinations
Cardiac Magnetic Resonance Fingerprinting	Neural networks for dictionary-free reconstruction	- Simultaneous acquisition of T1/T2 mapping - Potential to map other tissue characteristics (e.g., perfusion/scar)	cMRF:
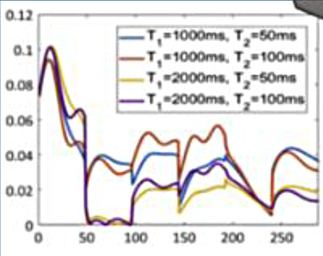			- Single acquisition for multiple tissue characteristics - Robustness against cardiac rhythms - Potential for standardization across scanner vendors. - Only in proof-of-concept stage
Diffusion Tensor Cardiac MRI	Accelerated acquisition through CNN based denoising and diffusion-tensor quantification from undersampled data (reduced averaging/repetitions required)	- Contrast-free detection of microstructural alteration (e.g., subclinical HCM, post-MI remodelling)	AI denoising and reconstruction
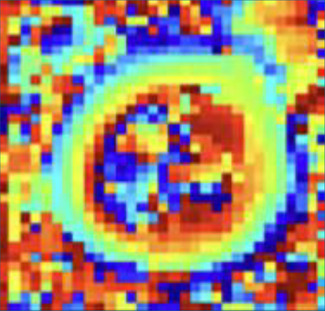			- Overcomes intrinsic low signal-to-noise ratio (SNR) encountered by the technique - Improves acquisition speed and therefore clinical translation
Radiomics and Deep-learning feature extraction	Unsupervised ML methods (clustering, principal component analysis, support vector machines) Combined modalities for higher-dimensional characterisation e.g., wall thickening/ strain + texture analysis Combined deep-learning and radiomics features Direct analysis of CMR using 2D/3D CNNs or vision transformers	- Disease discrimination [e.g., HCM vs. hypertension (HTN)] - Risk stratification - Detection of subtle tissue changes [e.g., chronic inflammation in dilated cardiomyopathy (DCM)] - Texture analysis-based contrast-free rule-out of LGE - Integration with phenome wide-associations in large-scale population studies	Radiomics
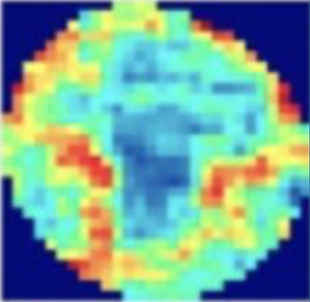			- Conventional CMR can be used—no additional acquisition required - Challenge: domain shift caused by influence of scanner type, vendor and sequence parameters in training - Larger prospective studies are needed
			Vision neural networks
			- Lack of large labelled datasets for training and validation

CNN, convolutional neural networks; FCN, fully convolutional networks; LSTM, long short-term memory; RNN, recurrent neural networks; GradCAM, Gradient weighted Class activation mapping; HCM, hypertrophic cardiomyopathy; ICM, ischaemic cardiomyopathy; LGE, late gadolinium enhancement; GAN, generative adversarial network; VNE, virtual native enhancement; CGE, cine generated enhancement; MI, myocardial infarction; MOCO, motion correction; DL, deep learning; cMRF, cardiac magnetic resonance fingerprinting; SNR, signal to noise ratio; ML, machine learning; HTN, hypertension; DCM, dilated cardiomyopathy; ECV, extracellular volume; vECV, virtual extracellular volume.

**Table 2:** Summary of the studies cited.

Study	Aims	Methods	Main findings
Late Gadolinium enhancement segmentation			
Fahmy et al. (14)	- Develop and evaluate performance of automated LGE segmentation in patients with HCM	- 3D CNN - Train *n* = 866, test *n* = 207 - Multi-site, multi-vendor - Stratified internal validation - Compared to manual quantification & 2D CNN	- Rapid acquisition (0.15s per image) - Good agreement with manual LGE quantification - Outperformed 2D CNN for agreement with manual LGE
Ghanbari et al. (18)	- Develop and evaluate automated LGE segmentation in patients with IHD	- FCN - Train *n* = 535, test *n* = 246 - Internal validation - Compared to manual quantification	- Good agreement with manual LGE quantification - Outperformed clinicians in predicting arrhythmic events
Moccia et al. (19)	- Test feasibility of automated LGE segmentation of infarcts - Compare whole LGE images vs. LV only input	- FCN - Train/test *n* = 30 (leave-one-patient-out cross-validation LOPO-CV) - Internal validation - Compared to manual quantification	- Feasible for LGE detection - Limiting search area to LV improved performance
Cui et al. (20)	- Perform unsupervised LGE segmentation by leveraging labelled cine images through domain adaptation	- Train a cine-labelled segmentation network and adapt it to LGE by aligning their image features using a variational autoencoder-based unsupervised domain adaptation framework	- Technique improved unsupervised LGE segmentation - Outperformed existing methods tested across public datasets
Lalande et al. (29)	- eMEDIC challenge results described	- A contest where several CNNs evaluated for discrimination between LGE images with & without infarct & extent of infarct	- CNN accurately discriminates infarct from non-infarct - Segmentation of areas of infarct remains challenging
Jacob et al. (30)	- Perform scar burden quantification for detecting myocardial pathologies (normal, dilated, hypertrophic, ischaemic)	- Foundational model pre-trained on millions of unlabelled images - DL method, train *n* = 159, test *n* = 53 - external validation *n* = 662	- Scar segmentation model trained without labelling is feasible - Clinically valuable
Parametric mapping and synthetic post-contrast imaging			
Zhang et al. (1)	- In patients with HCM - Generate LGE-like scar images from non-contrast images (native T1 maps + cines), termed virtual native enhancement (VNE)	- Conditional generative adversarial network: - Train *n* = 1,075 (QC’d), test *n* = 121 (QC'd) - internal validation - Blinded assessors graded the image quality of LGE and VNE and quantified both using standard techniques	- VNE images had better quality than LGE images. - VNE scar had good visuospatial and quantitative agreement with LGE
Zhang et al. (2)	- In patients with chronic MI - Generate LGE-like scar images from non-contrast images (native T1 maps + cines), termed virtual native enhancement	- Conditional generative adversarial network: - Train *n* = 775 (QC'd), test *n* = 68 (QC'd) - internal validation - Blinded assessors graded the image quality of LGE and VNE and quantified both using standard techniques	- VNE: 84% accuracy in detecting MI (LGE ground truth), 100% specificity - Better image quality than LGE - Good agreement in infarct quantification and transmurality - Validated in porcine models
Qi et al. (16)	- In patients with acute MI - Generate and evaluate LGE-like scar images from cine images (CGE-cine-generated enhancement)	- Generative adversarial network - Train *n* = 289, test *n* = 52/40 (internal/external) - CGE images were compared with LGE for quality using blinded observers and scar quantification (CGE/LGE using standard manual techniques)	CGE: - Superior image quality to LGE - Accurate scar quantification compared to LGE ground truth
Xu et al. (21)	- In patients with suspected MI - Detect MI from non-contrast cine MRI by learning abnormal motion patterns	- DL combined LV-focused ROI cropping - Local spatiotemporal (LSTM) and global optical-flow motion features - Train/test *n* = 165, (split not specified), internally validated - LGE-segmented infarct as the gold standard provided by two expert radiologists.	- Good accuracy compared to manual ground truth
Gonzales et al. (31)	- Motion artefact correction in native T1 maps	- DL-MOCO CNN - Train *n* = 1,536 UK Biobank with motion artefacts artificially generated, test *n* = 200 with motion - Internal validation. - DL MOCO compared to standard image registration	- Fast (<1s per T1 map) - Suppressed a wide range of motion artefacts - Better MOCO compared to traditional methods.
Felsner et al. (11)	- To assess an end-to-end DL algorithm to accelerate free-breathing 3D whole heart joint T1/T2 mapping.	- Non-rigid motion-corrected reconstruction network was used to estimate reconstructions of highly undersampled data - Train *n* = 51, test = 6—random split with 10-fold cross-validation. Internal validation.	- Highly accelerated MOCO reconstruction (370x) - Good agreement with reference standard (HDPROST)
Nowak et al. (27)	- Generate contrast-free virtual ECV (vECV) from native T1 maps to discriminate disease (myocarditis/amyloidosis) from health	GAN - Train *n* = 88; internal test set: *n* = 123 - External validation *n* = 96 - vECV was compared against true ECV values and assessed for diagnostic performance in myocarditis and amyloidosis	- vECV: good discrimination - Strong agreement between quantification in vECV and true ECV in normal studies and myocarditis - Limited quantitative agreement in amyloidosis
Cardiac Magnetic Resonance Fingerprinting			
Hamilton et al. (22)	- DL to rapidly reconstruct T1 and T2 maps from undersampled ECG-triggered cMRF data.	- CNN trained to output T1/T2 from cMRF signal-time course + RR intervals - Train: 8 million signals across 4,000 cardiac rhythms, test *n* = 58 healthy volunteers using Monte-Carlo simulations (1.5 T)	- Low error (robust) - Good *in-vivo* agreement with standard technique (dictionary matching) - 700x acceleration
Eck et al. (34)	- cMRF for rapid, simultaneous myocardial T1/T2 mapping to detect cardiac amyloidosis	- Prospectively gated 3 T cMRF; tissue classification using linear discriminant analysis (LDA) applied to either native T1/T2 or full cMRF signal timecourses - Study cohort: 9 cardiac amyloidosis patients, 5 controls	- Elevated myocardial T1 and T2 in CA vs. controls - Signal-timecourse-based LDA showed markedly improved group separability compared to native T1/T2
Cavallo et al (35)	- CMR Fingerprinting (cMRF) for simultaneous myocardial T1, T2, and ECV quantification in non-ischaemic cardiomyopathy	- Evaluated in patients with nonischemic cardiomyopathy vs. controls	- Demonstrated feasibility of joint T1/T2/ECV quantification in a clinical cohort
Diffusion Tensor Imaging			
Phipps et al. (15)	- Accelerate DTI by reducing signal averaging in participants living with obesity	- Residual denoising CNN - Tested on 20 healthy volunteers, 6 with obesity - DTI reconstructed using 8 averages (reference standard) and accelerated: 4, 2, and 1 average(s) - image quality and DTI parameters compared-train, *n* = 10 healthy volunteers (23,040 images)	- DL reconstructed 4 average no different to 8 average in image quality and DTI parameters - Differences between health and patients with obesity were preserved - 2x acceleration
Ferreira et al. (23)	- Accelerate DTI and reduced breath-holds (BH) for acquisition	- U-net reconstruction - DTI parameters predicted from reduced diffusion-weighted acquisitions (5BH, 3BH, 1BH) - Train *n* = 520, validation 112, test *n* = 122 - DL performance compared to reference standard (LLS) conventional tensor fitting	- Small differences in DTI parameters between LLS and U-Net methods - U-Net outperformed LLS - for reduced datasets - U-net preserved clinically relevant metrics with fewer-breath-holds
Wang et al. (38)	- Correct interframe motion in DTI	- Unsupervised DL framework - Total dataset (*n* = 948) - Trained by optimising a registration objective directly on the data (no ground truth) - Tensor aware cascade alignment correcting in-plane and through-plane motion - Compared three traditional and two DL methods	- Improved tensor accuracy with DL - Best helix-angle agreement with DL - Rapid execution
Radiomics and Deep-learning feature extraction			
Neisus et al (40)	- Differentiate hypertensive heart disease (HHD) from HCM - Radiomics-based texture analysis (no deep learning)	- Handcrafted texture features extracted from native T1 maps - Classifier: support vector machine (SVM) - Cohort: *n* = 232 (HHD *n* = 53; HCM *n* = 108; controls *n* = 71) - Train/test split: 4:1 within each disease group	- Radiomics outperformed global native T1 in discrimination between HHD from HCM
Fan et al. (42)	- Differentiate area-at-risk (AAR) from infarct and remote myocardium in Acute MI - Radiomics-based texture analysis	- Handcrafted texture features extracted from T2-mapping - Cohort: reperfused AMI patients (*n* = 106); follow-up CMR in *n* = 45	- Texture features outperformed mean T2 for distinguishing AAR from infarct and remote zones - No association with functional recovery (EF, strain, LV remodelling)
Fahmy et al. (17)	- Screen for scar absence in HCM (to avoid unnecessary GBCA)	- CNN based feature extraction from bSSFP cines - Comparison of radiomics vs. DL vs. DL-radiomics combined - Train + internal test (*n* = 759) - External validation (*n* = 100)	- DL-Radiomics outperformed DL only and Radiomics only for discriminating scar absence - Overall moderate discrimination only - Improved model performance required before clinical utility
Nakamori et al. (24)	- Investigate whether CMR radiomics can distinguish between non-collagen and inflammation from collagen in DCM.	- Radiomics-based classification framework - Handcrafted (no DL) feature extraction from native T1, ECV and LGE - Dimensionality reduction using PCA to derive principal radiomics - Biopsy validated in DCM (*n* = 132)	- Radiomics outperformed T1/ECV for distinguishing non-collagenous vs. mild-moderate collagen expansion - Radiomics associated with inflammatory phenotype not detected by conventional CMR
Xiang et al. (25)	- Explore risk stratification after reperfused STEMI using radiomics applied to conventional ECV maps	- Supervised radiomics-based prognostic model - Handcrafted radiomics features (no DL) - Training (*n* = 2,347), external test cohort (*n* = 94)	ECV-Radiomics-based scoring outperformed conventional metrics for MACE prediction - Incremental to clinical markers
Raisi-Estabragh et al. (43)	- Estimate biological heart age using radiomics	- Handcrafted radiomics features capturing ventricular shape and myocardial texture - Bayesian ridge regression with 10-fold cross validation - UKB (*n* > 29,000) - Heart age = predicted age—chronological age	- Sex-specific radiomics associated with heart age - Phenome-wide association with obesity, cardiometabolic risk, multimorbidity and socioeconomic factors
Inacio et al. (44)	- Estimate biological heart age from cardiac motion	- Supervised DL using graph neural networks (cardiac surface motion modelled as a graph over time) - Train: *n* = 5,064—UKB	- GNN outperformed dense neural network and boosting models - Improved age prediction accuracy
Mancio et al. (41)	- Identification of HCM patients at low likelihood of LGE to enable avoidance of GBCA	- Cine-derived radiomics combined with regional wall thickness and thickening using XGBoost ML - Training *n* = 882, independent multicentre external validation *n* = 217	- High negative predictive value for LGE - Supporting a cine-only rule-out strategy in HCM when combined with radiomics
Challenges of translating current AI-enabled methods of tissue characterisation into clinical practice			
Puyol-Anton et al. (48)	- Assess sex and racial bias in AI segmentation of cine CMR	- CNN based automated segmentation tool (train ∼4k) for biventricular volumes, mass and EF assessed - Bias analysis by Dice scores and volumes errors	- Racial bias detected - Not explained by confounders
Zhang et al. (50)	- Develop automated quality control for T1 mapping	- CNN to detect motion artefacts on T1 maps - Attention supervision to focus the network on artefactual segments - Trained *n* = 2,568	- CNN outperformed human artefact detection
Augusto et al. (53)	- Develop automated MWT measurement in HCM (Laplace WT estimation)	2D CNN - Train *n* = 1,923 (multicentre multi-disease), test *n* = 60. External validation. - Data was compared to measurements of MWT made by 11 experts.	- ML superior MWT precision (test: re-test) compared with clinician experts.
Xue et al. (54)	- Automated inline (during scan acquisition) myocardial perfusion segmentation	- CNN model was trained to segment the LV, myocardium and RV on perfusion scans. - Train *n* = 1,034, test *n* = 200. External validation - Model outputs were compared with manual segmentation.	- High ML segmentation accuracy - Real-time inference (<1s)

LGE, late gadolinium enhancement; CNN, convolutional neural networks; IHD, ischaemic heart disease; FCNN, fully convolutional neural networks; LV, left ventricle; DL, deep learning; HCM, hypertrophic cardiomyopathy; QC, quality control; VNE, virtual native enhancement; CGE, cine generated enhancement; ROI, region of interest; LSTM, long short-term memory; MOCO, motion correction; HDPROST, High-Dimensional Patch-based Reconstruction using Optimized Similarity Thresholding; vECV, virtual extracellular volume; cMRF, cardiac magnetic resonance fingerprinting; LLS, linear least squares;, BH, breath-hold; GBCA, gadolinium based contrast agent.

### Challenges in tissue characterisation CMR and the promise of AI

Challenges remain across several stages of workflow in tissue characterisation CMR. Reliance on GBCA excludes certain patients, including those with severe renal impairment, needle phobia, or contrast allergy (1, 2). Image quality is variable, often degraded by cardiac and respiratory motion, and some techniques are inherently low-signal or require long acquisition and reconstruction times, increasing resource demands. Manual segmentation to delineate and quantify scar in LGE is time-consuming and prone to observer variability (14).

Artificial intelligence (AI) offers several advantages to address these challenges. AI can exploit and enhance native signals in non-contrast imaging to generate contrast-free scar mapping; automate labour-intensive tasks throughout the imaging pipeline including image acquisition, reconstruction and segmentation; align (register) images to address cardiac and breathing motion; enhance signal and resolution in under-sampled low-signal-to-noise datasets; and automate and enable novel feature extraction and predictive modelling (1, 2, 11, 14–27).

Most existing reviews of AI in CMR have centred on automation or acceleration of cardiac function assessment and general image analysis. In this review, we focus specifically on AI applied to left ventricular tissue characterisation (Table 1).

### Late gadolinium enhancement segmentation

LGE interpretation currently relies on expert identification of abnormal hyperintense regions and, as such, is time consuming, suffers from high inter-observer variability and is challenging due to heterogenous acquisition and analysis techniques; deep-learning AI has shown potential in overcoming this challenge.

In HCM, a three-dimensional convolutional neural network (CNN) showed good agreement with manual quantification, providing segmentation at high speed and maintained high performance across multiple scanner vendors (14). Automated LGE quantification of ischaemic scar using CNNs surpassed clinicians in prediction of arrhythmic events in an ischaemic cardiomyopathy cohort (18).

Other neural network architectures that have been explored for LGE segmentation include fully convolutional networks (FCNs) (19) and autoencoders (20). FCNs have been less explored than CNNs but have demonstrated good accuracy in a small study on ischaemic scar (19). Autoencoders have been applied to align features between cine (bSSFP) and LGE images thereby enabling more accurate scar segmentation (where annotations are sparse) by leveraging well annotated cine CMR (20). Another approach includes slice-level identification of the presence of scar accompanied by probabilistic scar localisation using interpretability techniques such as GradCAM (28).

The inclusion of LGE segmentation tasks in international medical imaging challenges has spurred the development of benchmarked segmentation and classification methods for this application (29). Moreover, LGE segmentation performed using a vision foundational model pretrained on millions of unlabelled images has shown promising performance and a potential means to overcome the shortage of large labelled LGE datasets (30).

However, a challenge in developing AI methods for LGE segmentation is the lack of standardised analysis criteria that can be used as ground truth for AI models. Large scale external validation of these methods is also required before widespread adoption into clinical care.

### Parametric mapping

Parametric mapping techniques (T1, T2, T2* and ECV mapping) traditionally involve mathematical fitting of different cardiac images acquired with different acquisition parameters. Appropriate alignment (registration) is essential, as unaccounted cardiac or respiration motion will reduce image quality and parameter quantification accuracy. AI has been applied to mapping techniques such as T1 to improve motion correction. For example, CNN approaches like MOCOnet, trained on over 1,500 UK Biobank T1 maps with artificially generated motion artefacts, achieved rapid (<1 s) and robust suppression of artefacts in native T1 maps from 200 test subjects, outperforming traditional methods in both visual quality and reproducibility (31). More recently, deep learning–based end-to-end reconstruction frameworks have integrated motion estimation and correction into a single pipeline for 3D whole-heart T1/T2 mapping, reducing reconstruction times from hours to seconds while preserving quantitative accuracy (11). These techniques demonstrate how deep learning can enhance motion correction, enabling more rapid and accurate mapping quantification. Commonly motion correction is applied in-line for clinical scans, and therefore work is needed for deployment across scanners and vendors.

### Synthetic post-contrast imaging

Contrast-free “synthetic LGE” has been developed through the use of generative adversarial networks (GANs). Two leading techniques have been developed: “virtual native enhancement (VNE)” (1, 2), which has native T1 maps and cine MRI as inputs, and has been applied to chronic myocardial infarction (MI) (2) and HCM (1), and “cine-generated enhancement (CGE)”, which identifies LGE from cine MRI only and has been applied to acute MI (16). Both techniques demonstrated potential to detect the respective pathologies tested. Furthermore, infarct VNE has been validated *ex-vivo* in porcine models (2).

The success of the synthetic LGE methods suggests that enough information to identify scar or fibrosis is likely to exist in contrast-free images which may be conceptually challenging for CMR operators. This scar identification may be suitable for AI only and difficult for human operators. The propensity of GANs for hallucinations makes it critical to validate this concept in large diverse datasets, especially in the presence of poor image quality (often degraded due to patient factors) found in the clinical arena (1, 32).

An alternative to GANs to synthesise LGE from cine CMR is the analysis of local motion biomarkers (such as displacements and local strains) in cine MRI. Here, scar is identified due to its different biomechanical properties (e.g., stiffness) when compared to healthy myocardium. In a small study, a motion-feature learning framework based on long-short term memory (LSTMs) applied to cine CMR identified myocardial infarction, achieving a high accuracy when evaluated against the manual segmentation ground-truth (21).

Despite modest patient numbers used to train these models (1, 2, 16), initial proof-of-concept work could support contrast-free identification of scar. Ruling out scar may be useful to negate the use of GBCA in patients with low pre-test probability. Further, as opposed to replicating LGE, these techniques may even provide incremental information, giving additional trust to LGE findings or even detecting subtle abnormalities missed by LGE alone.

Moreover, as for LGE, GANs have also been used to generate virtual contrast-enhanced T1 maps using native (contrast-free) T1 map inputs for virtual ECV mapping (vECV). vECV showed good agreement with conventional ECV in healthy volunteers and myocarditis but was more modest in cardiac amyloidosis. Authors also noted some focal mapping abnormalities were not recapitulated using vECV and some lesions were “hallucinated” a known hazard of GAN based deep learning. Nevertheless, the study determined proof-of-principle for virtual ECV to expand this valuable diagnostic tool to patients otherwise precluded from GBCA and faster and cheaper CMR (27).

### Cardiac magnetic resonance fingerprinting

Cardiac magnetic resonance fingerprinting (MRF) is an advanced MRI approach that simultaneously characterises several MR parameters (e.g., T1, T2, T2*, proton density, fat fraction, flow parameters) using a different paradigm to conventional MRI (33). In MRF, the application of MR pulses is not designed to create a human-interpretable image, but instead to match the response of the tissue in each voxel to a pre-existing database (dictionary) of properties (33). This approach has several advantages over conventional mapping including inherent co-registration of all parameter maps, avoidance of confounding based on system hardware, sequence, heart rate and arrhythmia (22). This technique has shown its ability to discriminate health from disease in proof-of-concept work in cardiac amyloidosis (34) and also has shown feasibility in non-ischaemic cardiomyopathy (35).

Artificial intelligence can be used to optimise MRF acquisition sequence design and to perform dictionary generation, reconstructions and post-processing at a small fraction of the time of traditional MRFs (22). For example, neural network approaches to cardiac MRF have demonstrated good reproducibility, robustness to cardiac rhythm variability, and the ability to reconstruct quantitative maps in under 400 ms (22). This has potentially laid the foundations for accelerating development in other tissue characteristics such as focal fibrosis and perfusion, and more widespread clinical implementation (34). AI-based methods are likely to accelerate cardiac MRF and improve its practical feasibility, supporting its future adoption in routine clinical practice.

### Diffusion tensor cardiac MRI

Cardiac diffusion tensor imaging (cDTI) measures the diffusion of water within an imaging voxel thereby characterising the myocardial microstructural environment and microstructural alteration (36, 37). Its high sensitivity has been utilised to detect microstructural alteration in subclinical HCM (individuals with sarcomeric mutations but without overt left ventricular hypertrophy) and early adverse remodelling in acute MI (36). DTI is an inherently low-signal-to-noise technique as it relies on diffusion-induced signal dephasing. Signal averaging from multiple repeated raw images is therefore used to overcome this, but leads to long scan-times which reduces ability for clinical translation. Moreover, DTI is highly sensitive to motion (cardiac or respiratory).

Denoising convolutional neural networks have been developed to subtract noise from cardiac DTI, needing two-/ four-fold fewer signal averages while preserving image quality and accurate parametric differences between healthy volunteers and individuals with obesity—a challenging patient group in this domain due to lack of surface-coil proximity to the heart (15). Deep learning has also been applied to reconstruct quantitative maps from undersampled data, reducing the number of breath-holds required in DTI (23). Further, AI methods have also been used to correct inter-frame motion in cardiac DTI with promising results (38).

### Radiomics and deep-learning feature extraction

Radiomics is an image analysis framework that extracts voxel-level features (quantitative properties) to characterise tissue phenotypes. Radiomics features can include intensity-based statistics, spatial texture metrics, tissue morphological parameters, and features derived from the application of image filters. Feature extraction is typically preceded by segmentation, i.e., by the identification of the desirable regions of interest in the image. Machine learning algorithms are then employed to select and non-linearly combine radiomic features in the optimal combination for a given task (e.g., the identification of pathology).

Feature selection and dimensionality reduction are typically performed with unsupervised machine learning methods such as principal component analysis or minimum redundancy maximum relevance techniques. Classification can then be performed using machine learning techniques such as support vector machines or random forests. Proof-of-concept studies have shown potential applications in disease discrimination, risk stratification and non-contrast identification of scar (39). Texture analysis has been applied to T1 mapping to enhance discrimination between HCM and hypertensive heart disease beyond T1 mapping alone (40). Further work in HCM has demonstrated the potential to combine texture analysis with regional wall thickening derived from cine imaging to identify patients without focal fibrosis, thereby avoiding unnecessary GBCA exposure. A particular strength of this study was the use of multi-centre external validation, supporting its generalisability and scalability (41). Texture analysis applied to T2 mapping permitted visualisation of “area-at-risk” in reperfused MI—an ability historically restricted to LGE—but this parameter did not translate into prognostication of functional recovery at convalescence (42). Furthermore, radiomics analysis of ECV mapping in reperfused ST-segment elevation myocardial infarction (STEMI) demonstrated incremental prognostic value for adverse events beyond conventional markers and ECV alone, potentially reflecting discrimination between intramyocardial haemorrhage and myocardial necrosis, which exert divergent effects on ECV (25). These findings highlight the ability of radiomics-based texture analysis to capture tissue heterogeneity and disease biology that are not apparent on conventional imaging. This concept is further supported by a recent study validating radiomics features derived from T1 and ECV mapping against septal myocardial biopsy histology, demonstrating the detection of chronic myocardial inflammation in dilated cardiomyopathy (24).

An important advantage of radiomics is that it can use conventional CMR to build models that provide disease insights unavailable from conventional radiological analysis. For example, a UK Biobank study developed a heart-age estimation model using radiomics features as inputs and chronological age as the output, deriving a “delta-heart-age” that was then associated with multi-organ, metabolic, and socioeconomic markers in a phenome-wide analysis (43).

However, direct analysis of cardiac MRI using neural networks (NNs), such as 2D and 3D convolutional neural networks or vision transformers, has the potential to outperform machine learning methods based on radiomics features (44). This is because, given enough data, these architectures can extract powerful imaging features for clinical tasks, at the expense of human interpretability. A bottleneck to the implementation of these NN methods, which traditional radiomics approaches do not suffer from, is the lack of large labelled datasets for training and validation. Another approach in this area is the combination of deep learning features with radiomics ones (17). Further work is needed to explore the clinical applicability of these techniques, especially their accuracy for diagnostic purposes. A challenge in translating radiomics and NN methods is domain shift due to differences in scanner type, field strength, and sequence parameters, highlighting the need for reproducible feature selection and robust network design and training.

### Challenges of translating current AI-enabled methods of tissue characterisation into clinical practice

Despite the substantial advantages afforded by AI, several challenges in widespread adoption remain. A major barrier to AI model development is the shortage of well-curated datasets with reliable clinical labels. Foundational models—large AI models pretrained using self-supervised learning on unlabelled data—offer a promising strategy to address this limitation, as they can be adapted to multiple downstream tasks using comparatively small labelled datasets (30, 45) This framework also supports integration of multi-imaging and multi-modal data, including genomics and medical reports analysed using large language models, enabling more complex clinical learning tasks.

Nevertheless, model generalisability remains a key challenge. AI models are often trained on relatively small datasets and perform poorly in out-of-distribution settings, such as external validation cohorts. This is particularly problematic in CMR due to variation in scanner hardware, imaging protocols, and patient populations, which also complicates benchmarking across models. In addition, AI tissue characterisation models are frequently trained on research datasets with higher image quality than encountered in routine clinical practice, often excluding patients with arrhythmias, implantable devices, or limited breath-hold capacity. Ensuring training datasets capture real-world acquisition variability is therefore a priority. Access to diverse clinical data is further constrained by patient confidentiality concerns (46). Federated learning offers a solution, enabling collaborative model development across institutions without direct data sharing. In this paradigm, training occurs locally and only model parameters (gradients,weights) are shared and aggregated centrally (47). Data imbalance also raises fairness concerns, as models trained on skewed datasets may underperform in under-represented groups, including by race and sex, potentially exacerbating health disparities. For example, cine segmentation models trained on UK Biobank data—where over 80% of participants are White—perform less well in more diverse populations (48). Furthermore, it is possible to identify race from cine images due to areas outside the heart such as subcutaneous fat, leading to potential for misuse (26). Proposed mitigation strategies include improving dataset balance—although this may be challenging in rare diseases—as well as generative data augmentation and group-specific model training (48). Furthermore, outputs from deep learning models are often difficult for humans to interpret (“black box”), creating additional barriers to clinical adoption. Explainable artificial intelligence (XAI) methodologies can, in some circumstances, be employed to enhance user trust and are likely to feature in next-generation AI models applied to tissue characterisation (49). For example, saliency mapping and Grad-CAM can identify image regions that contribute most strongly to model predictions and have been applied to tasks such as quality control in T1 mapping (50) and LGE classification (28). However, as demonstrated in AI-ECG applications, improvements in explainability must be balanced against potential reductions in predictive performance (51).

Safe deployment of AI tools will also require adherence to evolving regulatory standards. The US Food and Drug Administration (FDA) has issued Good Machine Learning Practice (GMLP) guidelines to promote transparency, robustness, and quality control in medical AI systems (52). Importantly, regulatory frameworks may need to evolve further to accommodate adaptive or continuously learning AI tools, which differ fundamentally from static, “locked” algorithms. Successful integration of AI-based tissue characterisation into clinical workflows will depend on deployment through accessible open-source frameworks or seamless incorporation into vendor platforms, ensuring usability, interoperability, and clinician uptake. One promising approach is the real-time deployment of AI models during clinical MR image acquisition, enabling radiographers and clinicians to identify adverse features before the patient leaves the scanner bore, tailor imaging protocols, and reduce the need for repeat scans (53, 54). Such frameworks are also amenable to continuous learning through the ongoing acquisition of labelled clinical data, supporting iterative improvements in model performance.

### Future perspective

AI offers substantial advantages for tissue characterisation in CMR, with the potential to enhance diagnostic accuracy, improve risk modelling, and deepen disease understanding (Table 2). Direct clinical benefits include real-time quality control during image acquisition (55) and real-time detection of pathology. AI-based reconstruction using undersampling strategies can markedly accelerate acquisition and may be particularly impactful for low-field CMR systems, whose lower cost, reduced resource requirements, and improved safety profile offer a more scalable route to expanding access to cardiac MRI (56).

Future developments may include AI-driven co-registration of multiple CMR modalities—such as cines, LGE, DTI, and parametric maps—into a unified and more coherent three-dimensional representation. End-to-end deep learning approaches for probabilistic risk prediction from CMR images are also likely to expand, with explainable AI supporting interpretability and clinician trust. Finally, just as clinicians integrate clinical variables, ECG, and imaging to guide care, multimodal AI is expected to enable integration of these data at greater dimensionality and scale, supporting more accurate risk stratification and personalised therapy than previously possible. Clinicians alongside scientific and technical experts will be central to overseeing this evolution, ensuring fairness, generalisability, and robust performance for clinical care.

## References

[1] Zhang Q Burrage MK Lukaschuk E Shanmuganathan M Popescu IA Nikolaidou C Toward replacing late gadolinium enhancement with artificial intelligence virtual native enhancement for gadolinium-free cardiovascular magnetic resonance tissue characterization in hypertrophic cardiomyopathy. Circulation. (2021) 144(8):589–99. 10.1161/CIRCULATIONAHA.121.054432 34229451 PMC8378544

[2] Zhang Q Burrage MK Shanmuganathan M Gonzales RA Lukaschuk E Thomas KE Artificial intelligence for contrast-free MRI: scar assessment in myocardial infarction using deep learning–based virtual native enhancement. Circulation. (2022) 146(20):1492–503. 10.1161/CIRCULATIONAHA.122.060137 36124774 PMC9662825

[3] Kim RJ Wu E Rafael A Chen EL Parker MA Simonetti O The use of contrast-enhanced magnetic resonance imaging to identify reversible myocardial dysfunction. N Engl J Med. (2000) 343(20):1445–53. 10.1056/NEJM200011163432003 11078769

[4] Perera D Clayton T O’Kane PD Greenwood JP Weerackody R Ryan M Percutaneous revascularization for ischemic left ventricular dysfunction. N Engl J Med. (2022) 387(15):1351–60. 10.1056/NEJMoa2206606 36027563

[5] Ommen SR Ho CY Asif IM Balaji S Burke MA Day SM 2024 AHA/ACC/AMSSM/HRS/PACES/SCMR guideline for the management of hypertrophic cardiomyopathy: a report of the American heart association/American college of cardiology joint committee on clinical practice guidelines. Circulation. (2024) 149(23):e1239–311. 10.1161/CIR.0000000000001250 38718139

[6] Augusto JB Eiros R Nakou E Moura-Ferreira S Treibel TA Captur G Dilated cardiomyopathy and arrhythmogenic left ventricular cardiomyopathy: a comprehensive genotype-imaging phenotype study. Eur Heart J Cardiovasc Imaging. (2020) 21(3):326–36. 10.1093/ehjci/jez188 31317183

[7] Klem I Klein M Khan M Yang EY Nabi F Ivanov A Relationship of LVEF and myocardial scar to long-term mortality risk and mode of death in patients with nonischemic cardiomyopathy. Circulation. (2021) 143(14):1343–58. 10.1161/CIRCULATIONAHA.120.048477 33478245

[8] Flett A Cebula A Nicholas Z Adam R Ewings S Prasad S Rationale and study protocol for the BRITISH randomized trial (using cardiovascular magnetic resonance identified scar as the benchmark risk indication tool for implantable cardioverter defibrillators in patients with nonischemic cardiomyopathy and severe systolic heart failure). Am Heart J. (2023) 266:149–58. 10.1016/j.ahj.2023.09.008 37777041

[9] Messroghli DR Moon JC Ferreira VM Grosse-Wortmann L He T Kellman P Clinical recommendations for cardiovascular magnetic resonance mapping of T1, T2, T2* and extracellular volume: a consensus statement by the society for cardiovascular magnetic resonance (SCMR) endorsed by the European association for cardiovascular imaging (EACVI). J Cardiovasc Magn Reson. (2017) 19(1):75. 10.1186/s12968-017-0389-8 28992817 PMC5633041

[10] Ioannou A Patel RK Martinez-Naharro A Razvi Y Porcari A Hutt DF Tracking multiorgan treatment response in systemic AL-amyloidosis with cardiac magnetic resonance derived extracellular volume mapping. JACC: Cardiovasc Imaging. (2023) 16(8):1038–52. 10.1016/j.jcmg.2023.02.019 37178079 PMC10406611

[11] Felsner L Velasco C Phair A Fletcher TJ Qi H Botnar RM End-to-End deep learning-based motion correction and reconstruction for accelerated whole-heart joint T1/T2 mapping. Magn Reson Imaging. (2025) 121:110396. 10.1016/j.mri.2025.110396 40268172

[12] Richmann DP Contento J Cleveland V Hamman K Downing T Kanter J Accuracy of free-breathing multi-parametric SASHA in identifying T1 and T2 elevations in pediatric orthotopic heart transplant patients. Int J Cardiovasc Imaging. (2024) 40(1):83–91. 10.1007/s10554-023-02965-0 37874446 PMC10842347

[13] Carpenter JP Pennell DJ. Role of T2* magnetic resonance in monitoring iron chelation therapy. Acta Haematol. (2009) 122(2–3):146–54. 10.1159/000243799 19907152

[14] Fahmy AS Neisius U Chan RH Rowin EJ Manning WJ Maron MS Three-dimensional deep convolutional neural networks for automated myocardial scar quantification in hypertrophic cardiomyopathy: a multicenter multivendor study. Radiology. (2020) 294(1):52–60. 10.1148/radiol.2019190737 31714190 PMC6939743

[15] Phipps K van de Boomen M Eder R Michelhaugh SA Spahillari A Kim J Accelerated *in vivo* cardiac diffusion-tensor MRI using residual deep learning–based denoising in participants with obesity. Radiol Cardiothorac Imaging. (2021) 3(3):e200580. 10.1148/ryct.2021200580 34250491 PMC8259662

[16] Qi H Qian P Tang L Chen B An D Wu LM. Predicting late gadolinium enhancement of acute myocardial infarction in contrast-free cardiac cine MRI using deep generative learning. Circ Cardiovasc Imaging. (2024) 17(9):e016786. 10.1161/CIRCIMAGING.124.016786 39253820

[17] Fahmy AS Rowin EJ Arafati A Al-Otaibi T Maron MS Nezafat R. Radiomics and deep learning for myocardial scar screening in hypertrophic cardiomyopathy. J Cardiovasc Magn Reson. (2022) 24(1):40. 10.1186/s12968-022-00869-x 35761339 PMC9235098

[18] Ghanbari F Joyce T Lorenzoni V Guaricci AI Pavon AG Fusini L AI Cardiac MRI scar analysis aids prediction of Major arrhythmic events in the multicenter DERIVATE registry. Radiology. (2023) 307(3):e222239. 10.1148/radiol.222239 36943075

[19] Moccia S Banali R Martini C Muscogiuri G Pontone G Pepi M Development and testing of a deep learning-based strategy for scar segmentation on CMR-LGE images. Magn Reson Mater Phys Biol Med. (2019) 32(2):187–95. 10.1007/s10334-018-0718-4 30460430

[20] Cui H Li Y Wang Y Xu D Wu L-M Xia Y. Toward accurate cardiac MRI segmentation with variational autoencoder-based unsupervised domain adaptation. IEEE Trans Med Imaging. (2024) 43(8):2924–36. 10.1109/TMI.2024.3382624 38546999

[21] Xu C Xu L Gao Z Zhao S Zhang H Zhang Y Direct delineation of myocardial infarction without contrast agents using a joint motion feature learning architecture. Med Image Anal. (2018) 50:82–94. 10.1016/j.media.2018.09.001 30227385

[22] Hamilton JI Currey D Rajagopalan S Seiberlich N. Deep learning reconstruction for cardiac magnetic resonance fingerprinting T_1_ and T_2_ mapping. Magn Reson Med. (2021) 85(4):2127–35. 10.1002/mrm.28568 33107162 PMC10250104

[23] Ferreira PF Banerjee A Scott AD Khalique Z Yang G Rajakulasingam R Accelerating cardiac diffusion tensor imaging with a U-net based model: toward single breath-hold. J Magn Reson Imaging. (2022) 56(6):1691–704. 10.1002/jmri.28199 35460138 PMC9790699

[24] Nakamori S Amyar A Fahmy AS Ngo LH Ishida M Nakamura S Cardiovascular magnetic resonance radiomics to identify components of the extracellular matrix in dilated cardiomyopathy. Circulation. (2024) 150(1):7–18. 10.1161/CIRCULATIONAHA.123.067107 38808522 PMC11216881

[25] Xiang JY Dai YS Zheng JY Yu LY Hu J Song A Cardiac magnetic resonance-derived extracellular volume radiomics in reperfused ST-elevation myocardial infarction: long-term prognostic value and risk stratification. Eur Heart J Cardiovasc Imaging. (2025) 26(8):1376–86. 10.1093/ehjci/jeaf140 40338050

[26] Lee T Puyol-Antón E Ruijsink B Roujol S Barfoot T Ogbomo-Harmitt S An investigation into the causes of race bias in artificial intelligence–based cine cardiac magnetic resonance segmentation. Eur Heart J Digit Health. (2025) 6(3):350–8. 10.1093/ehjdh/ztaf008 40395422 PMC12088717

[27] Nowak S Bischoff LM Pennig L Kaya K Isaak A Theis M Deep learning virtual contrast-enhanced T1 mapping for contrast-free myocardial extracellular volume assessment. J Am Heart Assoc. (2024) 13(19):e035599. 10.1161/JAHA.124.035599 39344639 PMC11681454

[28] On Y Galazis C Chiu C Varela M. Two-Stage nnU-net for automatic multi-class bi-atrial segmentation from LGE-MRIs. In: Camara O Puyol-Antón E Sermesant M Suinesiaputra A Zhao J Wang C, editors. Statistical Atlases and Computational Models of the Heart Workshop, CMRxRecon and MBAS Challenge Papers. Cham: Springer Nature Switzerland (2025). p. 200–8.

[29] Lalande A Chen Z Pommier T Decourselle T Qayyum A Salomon M Deep learning methods for automatic evaluation of delayed enhancement-MRI. The results of the EMIDEC challenge. Med Image Anal. (2022) 79:102428. 10.1016/j.media.2022.102428 35500498

[30] Jacob AJ Sharma P Rueckert D. LGE Scar quantification using foundation models for cardiac disease classification. In: Jeong WK Kim HJ Deng Z Shen Y Aviles-Rivero AI Zhang S, editors. Foundation Models for General Medical AI. Cham: Springer Nature Switzerland (2026). p. 120–9.

[31] Gonzales RA Zhang Q Papież BW Werys K Lukaschuk E Popescu IA MOCOnet: robust motion correction of cardiovascular magnetic resonance T1 mapping using convolutional neural networks. Front Cardiovasc Med. (2021) 8:768245. 10.3389/fcvm.2021.768245 34888366 PMC8649951

[32] Neubauer S Kolm P Ho CY Kwong RY Desai MY Dolman SF Distinct subgroups in hypertrophic cardiomyopathy in the NHLBI HCM registry. J Am Coll Cardiol. (2019) 74(19):2333–45. 10.1016/j.jacc.2019.08.1057 31699273 PMC6905038

[33] Liu Y Hamilton J Rajagopalan S Seiberlich N. Cardiac magnetic resonance fingerprinting. JACC: Cardiovasc Imaging. (2018) 11(12):1837–53. 10.1016/j.jcmg.2018.08.028 30522686 PMC6394856

[34] Eck BL Seiberlich N Flamm SD Hamilton JI Suresh A Kumar Y Characterization of cardiac amyloidosis using cardiac magnetic resonance fingerprinting. Int J Cardiol. (2022) 351:107–10. 10.1016/j.ijcard.2021.12.038 34963645 PMC8857016

[35] Cavallo AU Liu Y Patterson A Al-Kindi S Hamilton J Gilkeson R CMR Fingerprinting for myocardial T1, T2, and ECV quantification in patients with nonischemic cardiomyopathy. JACC: Cardiovasc Imaging. (2019) 12(8_Part_1):1584–5. 10.1016/j.jcmg.2019.01.034 31103583

[36] Das A Kelly C Teh I Stoeck CT Kozerke S Sharrack N Pathophysiology of LV remodeling following STEMI: a longitudinal diffusion tensor CMR study. JACC: Cardiovasc Imaging. (2023) 16(2):159–71. 10.1016/j.jcmg.2022.04.002 36412993 PMC9902278

[37] Joy G Kelly CI Webber M Pierce I Teh I McGrath L Microstructural and microvascular phenotype of sarcomere mutation carriers and overt hypertrophic cardiomyopathy. Circulation. (2023) 148(10):808–18. 10.1161/CIRCULATIONAHA.123.063835 37463608 PMC10473031

[38] Wang F Luo Y Munoz C Wen K Luo Y Huang J Enhanced DTCMR with cascaded alignment and adaptive diffusion. IEEE Trans Med Imaging. (2025) 44(4):1866–77. 10.1109/TMI.2024.3523431 40030837

[39] Raisi-Estabragh Z Izquierdo C Campello VM Martin-Isla C Jaggi A Harvey NC Cardiac magnetic resonance radiomics: basic principles and clinical perspectives. Eur Heart J Cardiovasc Imaging. (2020) 21(4):349–56. 10.1093/ehjci/jeaa028 32142107 PMC7082724

[40] Neisius U El-Rewaidy H Nakamori S Rodriguez J Manning WJ Nezafat R. Radiomic analysis of myocardial native T1 imaging discriminates between hypertensive heart disease and hypertrophic cardiomyopathy. JACC: Cardiovasc Imaging. (2019) 12(10):1946–54. 10.1016/j.jcmg.2018.11.024 30660549 PMC7032053

[41] Mancio J Pashakhanloo F El-Rewaidy H Jang J Joshi G Csecs I Machine learning phenotyping of scarred myocardium from cine in hypertrophic cardiomyopathy. Eur Heart J Cardiovasc Imaging. (2022) 23(4):532–42. 10.1093/ehjci/jeab056 33779725 PMC9125682

[42] Fan ZY Wu Cw An DA Chen BH Wesemann LD He J Myocardial area at risk and salvage in reperfused acute MI measured by texture analysis of cardiac T2 mapping and its prediction value of functional recovery in the convalescent stage. Int J Cardiovasc Imaging. (2021) 37(12):3549–60. 10.1007/s10554-021-02336-7 34279752

[43] Raisi-Estabragh Z Salih A Gkontra P Atehortúa A Radeva P Boscolo Galazzo I Estimation of biological heart age using cardiovascular magnetic resonance radiomics. Sci Rep. (2022) 12(1):12805. 10.1038/s41598-022-16639-9 35896705 PMC9329281

[44] Inácio MH de A Shah M Jafari M Shehata N Meng Q Bai W Cardiac age prediction using graph neural networks. medRxiv. 2023.04.19.23287590. (2023).

[45] Bommasani R Hudson DA. Adeli E Altman R Arora S von Arx S Preprint TI—On the Opportunities and Risks of Foundation Models. arXiv e-prints. (2021).

[46] de Marvao A Dawes TJ Howard JP Regan O P D. Artificial intelligence and the cardiologist: what you need to know for 2020. Heart. (2020) 106(5):399. 10.1136/heartjnl-2019-316033 31974212 PMC7035692

[47] Rieke N Hancox J Li W Milletarì F Roth HR Albarqouni S The future of digital health with federated learning. npj Digital Medicine. (2020) 3(1):119. 10.1038/s41746-020-00323-1 33015372 PMC7490367

[48] Puyol-Antón E Ruijsink B Mariscal Harana J Piechnik SK Neubauer S Petersen SE Fairness in cardiac magnetic resonance imaging: assessing sex and racial bias in deep learning-based segmentation. Front Cardiovasc Med. (2022) 9:859310. 10.3389/fcvm.2022.859310 35463778 PMC9021445

[49] Salih A Boscolo Galazzo I Gkontra P Lee AM Lekadir K Raisi-Estabragh Z Explainable artificial intelligence and cardiac imaging: toward more interpretable models. Circ Cardiovasc Imaging. (2023) 16(4):e014519. 10.1161/CIRCIMAGING.122.014519 37042240

[50] Zhang Q Hann E Werys K Wu C Popescu I Lukaschuk E Deep learning with attention supervision for automated motion artefact detection in quality control of cardiac T1-mapping. Artif Intell Med. (2020) 110:101955. 10.1016/j.artmed.2020.101955 33250143 PMC7718111

[51] Patlatzoglou K Pastika L Barker J Sieliwonczyk E Khattak GR Zeidaabadi B The cost of explainability in artificial intelligence-enhanced electrocardiogram models. npj Digital Medicine. (2025) 8(1):747. 10.1038/s41746-025-02122-y 41350577 PMC12680752

[52] Artificial Intelligence/Machine Learning-enabled Working Group. Available online at: https://www.fda.gov/medical-devices/software-medical-device-samd/good-machine-learning-practice-medical-device-development-guiding-principles

[53] Augusto JB Davies RH Bhuva AN Knott KD Seraphim A Alfarih M Diagnosis and risk stratification in hypertrophic cardiomyopathy using machine learning wall thickness measurement: a comparison with human test-retest performance. Lancet Digit Health. (2021) 3(1):e20–8. 10.1016/S2589-7500(20)30267-3 33735065

[54] Xue H Davies RH Brown LAE Knott KD Kotecha T Fontana M Automated inline analysis of myocardial perfusion MRI with deep learning. Radiol Artif Intell. (2020) 2(6):e200009. 10.1148/ryai.2020200009 33330849 PMC7706884

[55] Cheung HC Vimalesvaran K Zaman S Michaelides M Shun-Shin MJ Francis DP Automating quality control in cardiac magnetic resonance: artificial intelligence for discriminative assessment of planning and motion artifacts and real-time reacquisition guidance. J Cardiovasc Magn Reson. (2024) 26(2):101067. 10.1016/j.jocmr.2024.101067 39079601 PMC11416635

[56] Campbell-Washburn AE Varghese J Nayak KS Ramasawmy R Simonetti OP. Cardiac MRI at low field strengths. J Magn Reson Imaging. (2024) 59(2):412–30. 10.1002/jmri.28890 37530545 PMC10834858

